# The effect of smaller classes on infection-related school absence: evidence from the Project STAR randomized controlled trial

**DOI:** 10.1186/s12889-023-17503-9

**Published:** 2024-01-03

**Authors:** Paul T. von Hippel

**Affiliations:** Center for Health and Social Policy, LBJ School of Public Affairs, University of Texas, Austin, USA

**Keywords:** Influenza, Infection, Class size

## Abstract

**Background:**

In an effort to reduce viral transmission, many schools reduced class sizes during the recent pandemic. Yet the effect of class size on transmission is unknown.

**Methods:**

We used data from Project STAR, a randomized controlled trial in which 10,816 Tennessee elementary students were assigned at random to smaller classes (13 to 17 students) or larger classes (22 to 26 students) in 1985-89. We merged Project STAR schools with data on local deaths from pneumonia and influenza in the 122 Cities Mortality Report System. Using mixed effects linear, Poisson, and negative binomial regression, we estimated the main effect of smaller classes on absence. We used an interaction to test whether the effect of small classes on absence was larger when and where community pneumonia and influenza prevalence was high.

**Results:**

Small classes reduced absence by 0.43 days/year (95% CI -0.06 to -0.80, *p* < 0.05), but small classes had no significant interaction with community pneumonia and influenza mortality (95% CI -0.27 to + 0.30, *p* > 0.90), indicating that the reduction in absence due to small classes was not larger when community disease prevalence was high.

**Conclusion:**

Small classes reduced absence, but the reduction was not larger when disease prevalence was high, so the reduction in absence was not necessarily achieved by reducing infection. Small classes, by themselves, may not suffice to reduce the spread of respiratory viruses.

**Supplementary Information:**

The online version contains supplementary material available at 10.1186/s12889-023-17503-9.

In response to the pandemic of COVID-19 (coronavirus disease 2019), caused by the virus SARS-CoV-2 (severe acute respiratory syndrome coronavirus 2), many schools implemented policies to reduce class size. During the 2020-21 school year, approximately one-half of US schools implemented some type of “hybrid” instruction policy that reduced in-person class size by having a substantial fraction of children stay home to receive instruction remotely [1]. European countries including France, Norway, Denmark, and Iceland restricted class size as a condition for reopening schools in person, and an OECD report described class size as a “critical parameter for the reopening of schools.” [2].

Concerns about class size resurfaced as schools contemplated reopening in late August 2021, when no COVID vaccine had yet been approved for elementary students (5- to 11-year-olds), only 40% of middle and high school students (12 to 17-year-olds) had been fully vaccinated [3] and only 61% of parents had received at least one vaccine dose [4].

The Biden administration, advised by the American Federation of Teachers (AFT), suggested hiring new teachers to reduce class sizes by approximately 10%. The $50 billion cost, the administration suggested, could be drawn from the $125 billion in federal funds allocated to public and private elementary and secondary schools under the American Rescue Plan of March 2021 [5, 6]. In April 2021, the US Department of Education endorsed reducing class size to “accommodate social distancing” and increase learning. Five states (Alaska, Minnesota, Maine, West Virginia, South Carolina) included class size reduction among their priorities for federal relief funds during the 2021-22 and 2022-23 school years; other states left the decision to districts [7].

The rationale for class size reduction is straightforward. With fewer children in a classroom, there is a lower probability that the classroom will contain an infected child, and fewer children to whom an infected child will be exposed. Children can sit or stand further apart, at least on average, reducing the chance of infection by direct contact or proximity. With fewer children to supervise, teachers and staff can devote more time and attention to disinfecting surfaces and enforcing healthy behaviors, such as wearing masks and washing hands. A simulation conducted in the first pandemic summer of 2020 predicted that reducing class sizes would substantially reduce COVID-19 outbreaks [8].

Within a few months, though, doubts arose about how much class size mattered for the spread of the novel coronavirus. Accumulating evidence suggested that COVID-19 did not spread primarily through direct contact or proximity to infected persons or surfaces, but through fine droplets and aerosol particles suspended in indoor air. It followed that class size was likely much less important than masks, which prevent many exhaled fine droplets from spreading into the air, and ventilation, which exchanges indoor air containing droplets and aerosols with less-contaminated outdoor air. A simulation predicted that if children talking in an indoor classroom did not wear masks, the risk of transmission would become unacceptable within an hour for any class size greater than 10; by contrast, if children wore masks, the risk would remain acceptable for at least 13 h even if the class size was as large as 30 [9, 10].

Empirically, though, evidence regarding the effect of class size on transmission is limited and contradictory. Two observational studies have estimated the effect of US school reopening policies during the pandemic—fully online, fully in-person, or hybrid—on the incidence of COVID-19 hospitalizations [11] and positive COVID-19 tests [12] in fall 2020. Although classes were smaller in schools that reopened in a hybrid fashion, both studies found that opening in a hybrid rather than in-person fashion had no significant effect in counties where the prevalence of COVID-19 before schools reopened was low. In counties where the prior prevalence of COVID-19 was high, the results were mixed; one study concluded that fully opening schools in-person accelerated the spread of the virus, [12] while the other reported ambiguous results that were sensitive to model specification [11].

Evidence on the effect of class size on infection is limited not just for the novel coronavirus (SARS-CoV-2), but also for more familiar pathogens such as influenza. While correlations between class size and infection are sometimes reported in observational studies, [13] only one prior study tried to estimate the causal effect of reducing class size on influenza-related absence [14]. By exploiting discontinuities induced by Japanese laws limiting class size, the study concluded that reducing Tokyo class sizes to 27 from an average of 32 would have substantially reduced the risk of school closures due to outbreaks during the flu seasons of 2015-2017 [11]. Below a class size of 27, the benefits of further reductions were less clear. Note that most US classes are already smaller than 27 students; average US class size is 17 to 26, depending on grade level and class type [15].

In this study, we estimate the effect on influenza-related absence of reducing average class size from 23 to 15. We use evidence from a randomized controlled trial that assigned young children to larger and smaller classes at random. We estimate the main effect of class size on absence, as well as the interaction between class size and community influenza prevalence. Our hypothesis is that if class size reduces influenza transmission, the effect of class size on absence should be larger in times and places when community influenza prevalence is high.

## Methods

### Project STAR randomized controlled trial

Our primary data come from Tennessee’s Student/Teacher Achievement Ratio Project (Project STAR)—a four-year block-randomized longitudinal trial of class size reduction, funded by a $12 million appropriation from Tennessee’s House Bill 544, which was passed by the Tennessee State Legislature in May 1985 [16–19].

According to Project STAR’s design, within each participating school children and teachers were assigned at random to three classroom treatments in kindergarten: (1) small classes with a target size of 13 to 17 students, (2) regular-sized classes with a target size of 22 to 26 students, or (3) regular-sized class with a teacher’s aide. Children were followed from kindergarten in 1985-86 through third grade in 1988-89. Children who spent kindergarten in a small class remained in small classes from kindergarten through third grade. Children who spent kindergarten in one of the other conditions—a regular-sized class or a regular-sized class with an aide—were re-randomized between those two conditions after kindergarten. Children who entered participating schools after kindergarten were randomized among the three conditions as well.

### Participants

Out of the 886 elementary schools in Tennessee, 180 volunteered for Project STAR, but only 100 of those schools were large enough to offer at least one kindergarten class in each of the experimental conditions—i.e., at least one classroom with 13 to 17 students and at least two classrooms with 22 to 26 students each [16]. After some negotiation, 79 schools were selected to participate. During the four years of Project STAR, four schools withdrew from the study, and students in one rural county progressed from a feeder school offering only kindergarten to a lower elementary school offering grades 1–3.

### Dependent measure

The original purpose of Project STAR was to estimate the effects of reducing class size on test scores in grades K-3; [17] later analyses also estimated effects on grade repetition, high school graduation, college attendance and completion, and early adult employment and wages [20, 21]. Ours is the first analysis of Project STAR to look at absence and its correlation with infectious disease prevalence.

Project STAR recorded the number of days that each student was absent in three of the four study years: kindergarten 1985-86, first grade 1986-87, and third grade 1988-89, but not second grade 1987-88. Project STAR did not record the reasons for absence, but past studies suggest that approximately half of school absences are due to illness [22–24]. Some efforts to estimate infection-related absence have relied on correlations between absence among schoolchildren and disease prevalence in the larger community [25]. That is the strategy that we adopt here.

### Supplemental data from the 122 cities mortality reporting system (CMRS)

To estimate the correlation between absence and infection, we merged Project STAR with data from the 122 Cities Mortality Reporting System (CMRS), a surveillance study run by the Centers for Disease Control and Prevention from 1962 to 2016 [26]. The CMRS recorded mortality data for 122 US cities, including the four largest cities in Tennessee: Memphis, Nashville, Knoxville, and Chattanooga. For some cities, the data include the surrounding metropolitan area; for others, they are limited to the city proper. Thirty-three of the 79 schools that started Project STAR were in cities covered by the CMRS (Table 1).

**Table 1:** Description of Project STAR randomized controlled trial, Tennessee, school years 1985-89

Year	1985-86	1986-87	1988-89	All 3 years
Grade	K	1	3	
Outcome				
Absences (mean)	10.5	7.6	6.8	8.3
Treatments (randomly assigned)				
% small class	30	28	32	30
% regular-sized class	35	38	31	34
% regular-sized class with teacher’s aide	35	33	38	35
Covariates				
% female	49	48	48	48
% free lunch	48	52	50	50
% white	67	66	66	66
% black	32	33	34	33
% other race/ethnicity (incl. Hispanic, Asian, Native American)	0.5	0.8	0.5	0.6
Sample size				
Observations	6,251	6,662	6,586	19,499
Distinct students	6,251	6,662	6,586	10,816
Teachers (classrooms)	325	337	331	993
Schools	79	76	74	80
Number of schools by district				
Memphis Public Schools	20	19	19	20
Knox County Public Schools (incl. Knoxville)	5	4	4	5
Davidson County Public Schools (incl. Nashville)	4	4	4	4
Hamilton County Public Schools (incl. Chattanooga)	3	3	3	3
Other school districts	47	46	44	48

For each city and week, the CMRS recorded the total number of deaths, as well as the number of deaths that were due to pneumonia and influenza (PI). Deaths were reported for each week, and we aggregated them to each school year. The school year was defined as running from week 34 of one calendar year to week 22 of the next. For example, deaths during the kindergarten school year of 1985-86 were defined as the total of deaths from week 34 of 1985 (starting August 24) through week 22 of 1986 (starting May 31). Changing the beginning and end of the school year by a few weeks would not materially change the results, since the vast majority of PI deaths were concentrated in December and January.

For each city and school year, we calculated PI mortality—the percentage of deaths that were due to PI. PI mortality is often interpreted as a proxy for the prevalence and virulence of influenza viruses [31]. It is far from a perfect proxy, since many people contract influenza without dying of it, yet it does have some validity since students miss school more often during weeks when PI mortality peaks [25]. Although we would have liked to have separate estimates of PI mortality for the attendance zone of each school, PI mortality was only available at the city level. This is a common limitation in infectious disease surveillance, which state agencies, federal agencies, and private insurers commonly aggregate to larger geographic areas such as cities, counties, or multi-county regions [11, 12, 27, 28].

To merge the CMRS with the Project STAR data, we had to identify the location of each Project STAR school. The Project STAR data do not identify schools explicitly, but we identified them by merging with other data. In particular, Table II in the Project STAR *Technical Report* [32] listed the name and district of all 80 participating schools, along with the number of small classes, regular classes, and regular classes with an aide that each school offered in each year of Project STAR. Other characteristics of Tennessee schools were available in the US Department of Education’s Common Core of Data, [33] which provided data on every US school back to 1986-87 (year 2 of Project STAR). By matching the Project STAR data to variables from the Technical Report and Common Core, we identified which schools in the Project STAR were in the four cities surveyed by the CMRS, and that gave us the community PI mortality for those schools. We used the *ultimatch* command for Stata to minimize the Euclidean distance between matched schools [34]. Alternative matching procedures yielded identical estimates; in the few cases where the matched school differed, the matched city was the same, so the matched value of community PI mortality, which was measured at the city level, did not change.

### Data analysis

Because Project STAR assigned children and teachers to treatments at random, we could estimate the effect of class size by comparing the average number of absences in each treatment group. To maximize our power to detect an effect, we pooled data longitudinally across the years of the study. In analyzing the pooled data, we accounted for correlations among observations of the same child in different years, as well as correlations among different children in the same classroom and school year.

More specifically, we fit the following linear mixed model:

$$\eqalign{ Absenc{e}_{ctsdg}& ={\alpha }_{s}+{\beta }_{g}+{\gamma }_{1}{Small}_{ctsdg} \cr &+{\gamma }_{2}Aid{e}_{ctsdg} +\dots +{u}_{c}+{e}_{ctsdg}}$$

Here $Absenc{e}_{ctsdg}$ was the number of absences for child *c* with teacher *t* in school *s* and district *d* during grade *g* (kindergarten 1985-86, first grade 1986-87, or third grade 1988-89). ${Small}_{ctsdg}$ and $Aid{e}_{ctsdg}$ indicated which experimental treatment the child received during that grade—a small class or a regular class with an aide; regular classes without an aide were the reference. ${\alpha }_{s}$ was a school fixed effect, which accounted for the fact that some schools had higher absence rates than others, and children were randomized to conditions within schools rather than between then. ${\beta }_{g}$ was a grade fixed effects, which accounted for the fact that absence rates were higher in kindergarten than in later grades. ${u}_{c}$ was a child random effect used to model the correlation among observations of the same child in different grades. ${e}_{ctsdg}$ is a random residual, clustered at the classroom level to account for the correlation among observations of different children in the same classroom. We estimated the model using the *xtreg* command in Stata software, version 16.1.

Because the experimental treatments were assigned at random, they were not correlated with any child characteristics, so no child-level covariates were needed to get unbiased estimates of treatment effects. Nevertheless, we fit the model both with and without covariates representing each child’s race, gender, and free lunch eligibility (an indicator of poverty). Unsurprisingly, these covariates changed the results very little.

To estimate whether the effect of class size on absence was stronger in communities and years with higher infection rates, we added a covariate $P{I}_{sg}$, representing PI mortality in district *d* during the school year when the child was in grade *g*. We centered $P{I}_{sg}$ around its mean of 7.3, and we let the mean-centered variable interact with the experimental treatments:

$$\eqalign{Absenc{e}_{ctsdg}& ={\alpha }_{s}+{\beta }_{g}+{\gamma }_{1}{Small}_{ctsdg}+{\gamma }_{2}Aid{e}_{ctsdg} \cr & +{\gamma }_{3}P{I}_{dg} +{\delta }_{11}P{I}_{dg}\times {Small}_{ctsdg} \cr & +{\delta }_{12}P{I}_{dg}\times Aid{e}_{ctsdg}+\dots +{u}_{c}+{e}_{ctsdg}}$$

Although community infection rates could not be randomized, the coefficients of $P{I}_{dg}$ and the interactions can be interpreted as causal effects if the year and school fixed effects control adequately for unobserved confounding variables that vary between schools and years. Again, we fit the model both with and without covariates for race, gender, and free lunch eligibility. Again, the covariates made little difference to the results.

Because absences is a count variable, we also fit Poisson and negative binomial models with the same fixed effects, random effects, and covariates. The results, given in the Online Supplement, were very similar.

## Results

Table 1 summarizes the design of Project STAR and the characteristics of participating children, schools, and school districts. The table reports on children with absence data in kindergarten, first, or third grade; no absence data was recorded in second grade. In each of those three school years, over 6,000 children participated in over 300 classrooms; across all three years, over 10,000 distinct children participated in nearly 1,000 classrooms. Approximately equal numbers of classrooms were assigned to the three experimental treatments, but because the small classrooms had fewer students, slightly less than one-third of children were assigned to the small class condition. Nearly two-thirds of participating children were white, one-third were black, and less than 1% were other races and ethnicities. Just under half of participating children were female, and precisely half were poor enough to receive free school lunches. Comparisons elsewhere show that Project STAR students were poorer and more likely to be black than children living elsewhere in Tennessee and other states in the 1980s [20].

The bottom of Table 1 shows the distribution of participating schools across Tennessee school districts. About one-third of participating schools were in the four cities covered by the CMRS. Fully a quarter of participating schools were in Memphis, while another 15% were in Knox County (principally Knoxville), Davidson County (principally Nashville), and Hamilton County (principally Chattanooga).

**Fig. 1:**
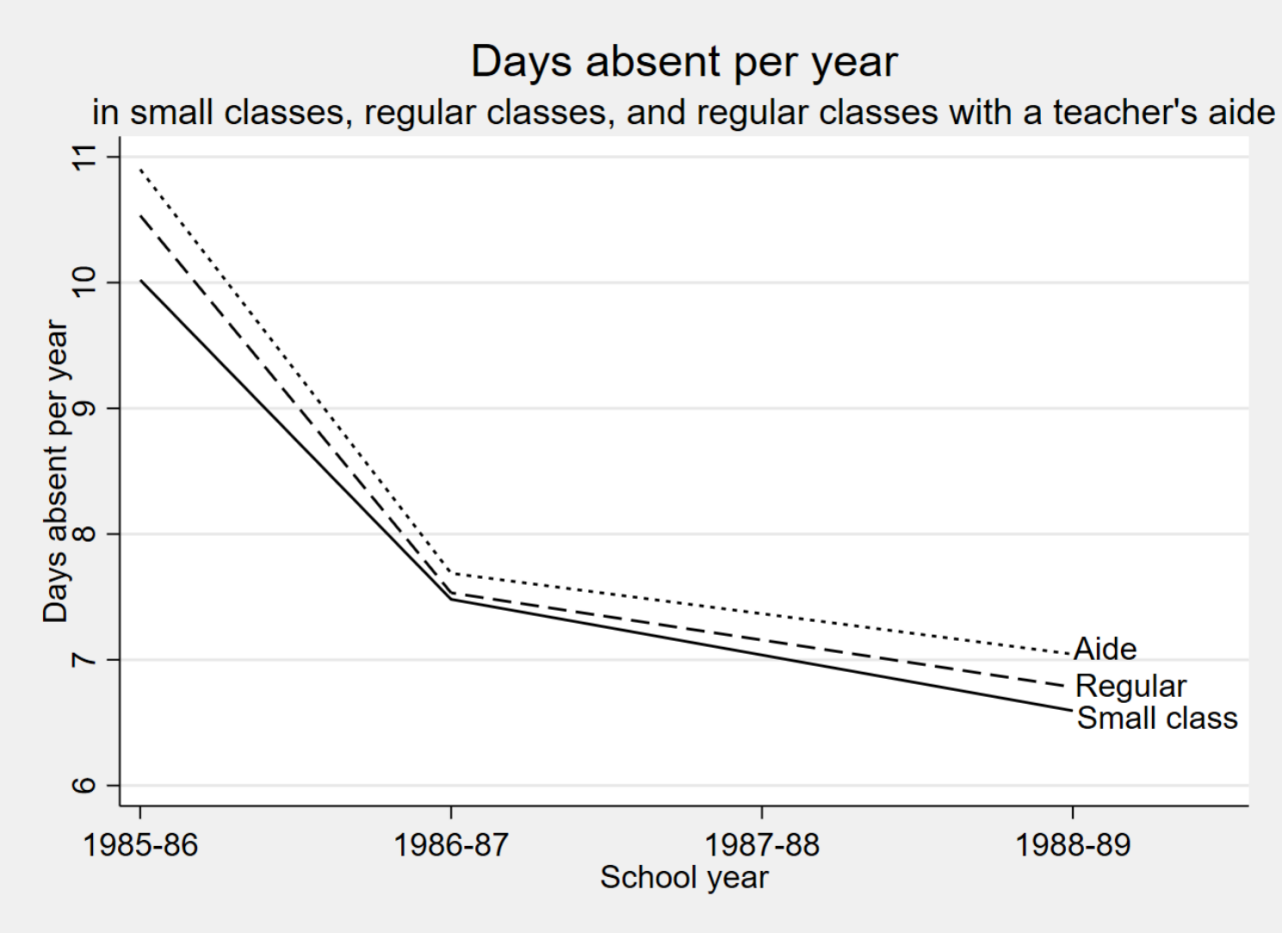
Smaller classes had slightly fewer days absent, on average, than regular-sized classes or regular-sized classes with a teacher’s aide

The top of Table 1 shows the average number of absences per student per year, which dropped from over 10 in kindergarten 1985-86 to less than 7 by third grade 1988-89. Figure 1 compares absences across the three experimental treatments. The differences were small but consistent across kindergarten, first, and third grade, with each year having fewer absences in small classes than in regular-sized classes or regular-sized classes with a teachers’ aide. Table 2 shows that these differences are statistically significant (*p* < 0.05), with smaller classes having 0.4 fewer annual absences per student, on average, across kindergarten, first, and third grade. Including gender, race, and free lunch status as covariates had practically no effect on this result.

**Table 2:** Linear mixed model predicting days of absence per year, Project STAR, Tennessee, school years 1985-89

c	Coef. (95% CI)	Coef. (95% CI)	Coef. (95% CI)	Coef. (95% CI)
Small class	-0.43* (-0.80, -0.06)	-0.42* (-0.79, -0.05)	-0.49+ (-1.01, 0.04)	-0.43 (-0.95, 0.09)
Teacher’s aide	0.21 (-0.13, 0.54)	0.20 (-0.14, 0.54)	0.20 (-0.28, 0.68)	0.19 (-0.29, 0.67)
First grade (ref. kindergarten)	-2.70** (-3.00, -2.41)	-2.73** (-3.03, -2.43)	-1.94** (-2.42, -1.46)	-1.97** (-2.44, -1.50)
Third grade (ref. kindergarten)	-3.33** (-3.64, -3.03)	-3.36** (-3.66, -3.06)	-3.38** (-4.39, -2.37)	-3.46** (-4.43, -2.48)
PI			0.38* (0.00, 0.75)	0.39* (0.02, 0.76)
Small class × PI			0.02 (-0.27, 0.30)	0.02 (-0.27, 0.30)
Teacher’s aide × PI			-0.00 (-0.28, 0.27)	-0.01 (-0.28, 0.26)
Female (ref. male)		0.26+ (-0.03, 0.55)		0.23 (-0.20, 0.65)
Black (ref. white)		-1.48** (-2.06, -0.90)		-1.19** (-2.06, -0.33)
Other race/ethnicity		-1.65* (-3.30, -0.00)		-2.10* (-3.83, -0.37)
Free lunch		1.64** (1.34, 1.93)		1.86** (1.40, 2.32)
Observations	19,499	19,329	8,073	8,028
Distinct children	10,816	10,726	4,966	4,936

*Note*. ** *p* < 0.01, * *p* < 0.05, + *p* < 0.1, two sided. Coef.=Regression coefficients. CI = classroom-clustered 95% confidence intervals. PI = percent of deaths due to pneumonia and influenza, mean-centered. All models include school fixed effects, year fixed effects, and child random effects

**Fig. 2:**
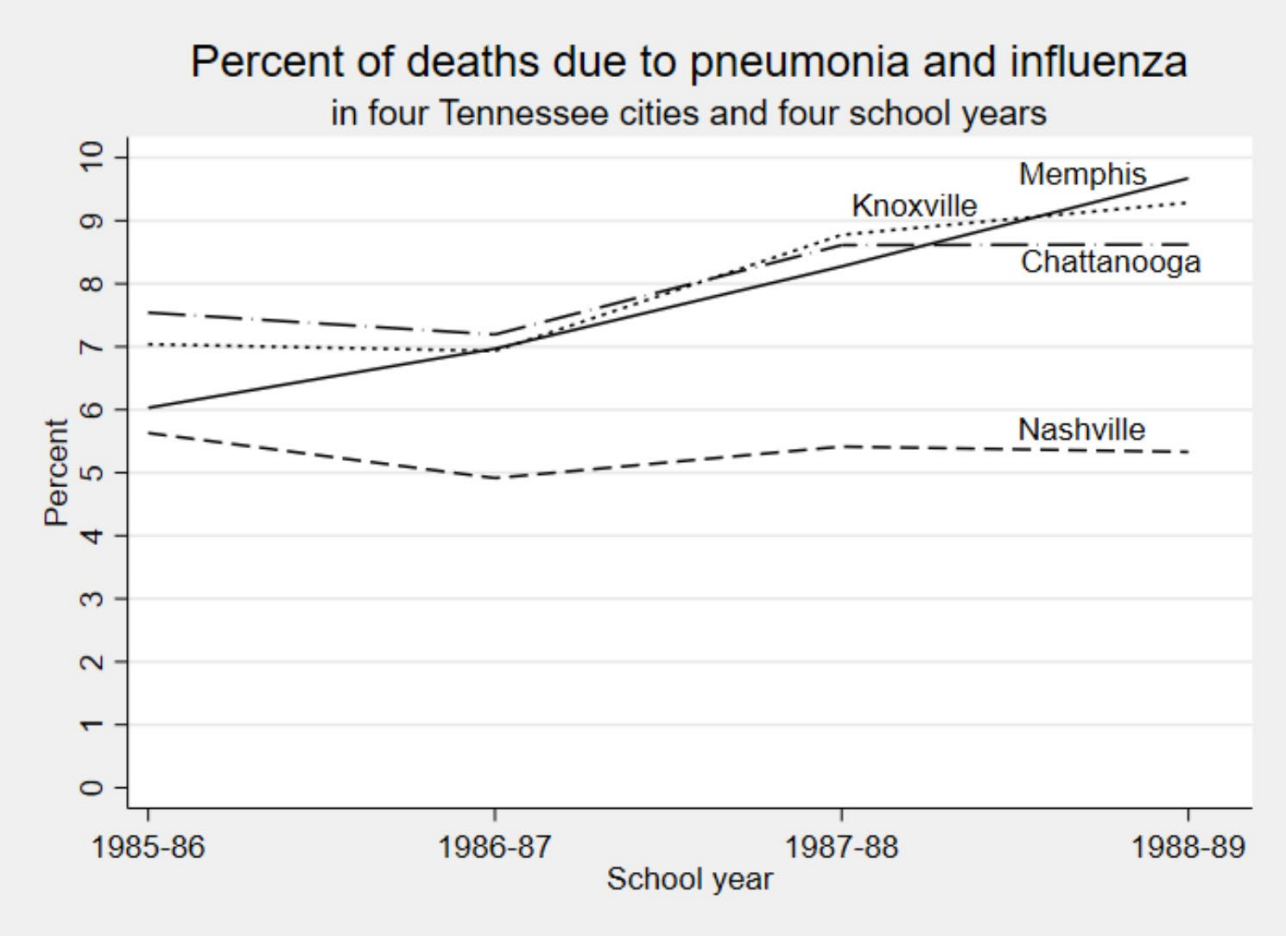
Percent of deaths due to pneumonia and influenza in four Tennessee cities and four school years

Although these results show that smaller classes reduced absence, it is not clear whether the reduction in absence was due to a reduction in infection. To address that question, we added community PI mortality to the model for the cities covered by the CMRS. Figure 2 summarizes trends in PI across the four cities. In Nashville, PI mortality held steady between 5 and 6% across the 4 years of Project STAR. In Memphis, PI mortality rose from 6 to nearly 10%, and in Knoxville and Chattanooga, PI mortality rose from approximately 7 to approximately 9%. The differences in levels and trends within and between cities help to identify the effect of PI mortality on absence.

Table 2 shows that PI mortality was a significant predictor (*p* < 0.05) of absence; a 1% point increase in PI mortality was associated with an increase of approximately 0.4 annual absences per student. However, community PI rates did not appear to moderate the effect of class size on absence. The interaction between PI and the small class size condition was close to zero and far from statistical significance (*p* > 0.9). The interaction between PI and the teacher’s aide condition was also close to zero and far from statistical significance (*p* > 0.9).

Including PI and interactions in the model slightly reduced the statistical significance of the main effect of small classes. The point estimate for the effect of small classes is similar in all models, but the confidence interval gets slightly wider and the *p* values get slightly larger when PI and interactions are included in the model (from *p* = 0.02 to *p* = 0.06 without covariates, from *p* = 0.03 to *p* = 0.11 with covariates). This is partly because the sample size is reduced to districts with PI data and partly because there is some correlation between the small class variable and its interaction with PI.

## Discussion

In Tennessee’s Project STAR randomized controlled trial, reducing class sizes by one-third significantly reduced annual absence by 0.4 days per child on average. Variation in absence rates were correlated with variation in PI mortality across cities and years, but the effect of class size on absence was not increased in years and communities with high PI mortality. Although smaller classes reduced absence, it is not clear that they did so by reducing infection.

### Strengths and limitations

This study has several strengths. One is that class size was assigned at random, guaranteeing that the association between class size and absence is causal. Another strength is the large sample size (over 10,000 children in 80 schools) and long study duration (4 years).

A final strength is the focus on influenza, a disease that affects school-age children strongly. School-age children infected with influenza often have symptoms so severe that they must stay home from school [29]—especially at the time of the study, in the 1980s, when influenza vaccines were not yet approved for healthy school-age children [30]. The highly symptomatic nature of influenza in school-age children should increase the correlation between absence rates and influenza infection, increasing the validity of absence as a proxy for infection.

Not only do school-age children suffer symptoms from influenza, schools also play a substantial role in transmitting influenza viruses [31]. During influenza pandemics, incidence spikes after schools open [32] and subsides, at least among school-age children, when schools close for two weeks or more [33, 34].

Yet our study’s focus on influenza in the 1980s also limits its relevance to the novel coronaviruses that began to spread in 2019. Unlike children infected with influenza, children infected by SARS-CoV-2 typically display mild or no symptoms [35]. While schools play a major role in transmitting influenza, they seem to play a comparatively small role in transmitting SARS-CoV-2. About half of COVID-19 studies have found no effect of school closures, and most studies found no effect of school reopenings on COVID-19 transmission [36].

Yet these differences between influenza and COVID-19 do not necessarily weaken our conclusions. Our finding that smaller classes did little to reduce absences that were correlated with influenza suggests that small classes might do even less to slow the spread of COVID-19, in which schools and school-age children play a smaller role.

A limitation of the data was that it did not distinguish between absences due to illness and absences due to other reasons. This is a common limitation, especially in older data, which rarely specified the reason for absence, at most reporting whether the absence was excused or unexcused [37]. Only a few recent studies from the United Kingdom have had data that distinguishes absence due to illness specifically [22, 37, 38]. Researchers with access to data on reasons for absence should examine the effect of class size on absence due to illness, especially in times and places where community infection prevalence is high.

An additional limitation of the data was its relatively limited geographic variation. Although the number of schools and districts was substantial, only four cities in the 122 Cities Mortality Report System were represented, and three of those cities had similar trends in PI mortality (Fig. 2). Future research on this topic should examine a wider variety of locations with more variation in disease prevalence.

### Policy implications

Project STAR did not take place during a pandemic, and although it reduced class size by 35% it did not change other practices. In this respect, the policy evaluated in Project STAR was different than the policies tried during the COVID-19 pandemic. During the COVID-19 pandemic, class size reductions of 50% or more were part of a multi-pronged strategy that included measures that were not used during Project STAR, such as mask-wearing, regular disinfection of surfaces, and avoidance of mass assemblies during recess and lunch.

Yet as the COVID-19 pandemic receded, the relevance of Project STAR to current policy increased. Many schools dropped mask mandates and resumed assemblies starting in 2021-22, yet federal funds continued to be available for class size reduction through September 2023.

Are current reductions in class size likely to reduce the spread of disease? Our results suggest that they may not. Project STAR reduced average class size by 35%, which is less than the 50% reductions that some hybrid schools enacted during the height of the pandemic, but far more than the 10% reduction that the White House suggested in its February 2021 proposal [5]. Yet we found little or no evidence that Project STAR reduced infection-related absence. It seems unlikely that the 10% reduction in class size suggested by the White House would do much to reduce the spread of COVID-19 or other pathogens.

Rather than spending $50 billion on class size reduction as the White House suggested, other uses of that money might do more to prevent the spread of disease. Examples consistent with CDC guidance [35] include resources and incentives for vaccination and testing of school staff, parents, and children; upgrading HVAC systems and running them longer and at higher volumes; and installing high-efficiency particulate air (HEPA) filters and ultraviolet germicidal irradiation (UVGI) systems [36].

### Electronic supplementary material

Below is the link to the electronic supplementary material.

Supplementary Material 1

## Data Availability

The Project STAR data and documentation are available in several places including, since 2008, the Harvard Dataverse (https://dataverse.harvard.edu/dataset.xhtml?persistentId=doi:10.7910/DVN/SIWH9F). A copy of the data and code used to produce results in this article are available at OSF (https://osf.io/scg6e/).
