# Supplementary material for: The effect of smaller classes on infection-related school absence: evidence from the Project STAR randomized controlled trial
Source: BMC Public Health. 2024 Jan 3;24:83. doi: 10.1186/s12889-023-17503-9 (PMC10765901; doi:10.1186/s12889-023-17503-9)
Supplement: Supplementary file 1 — Supplementary Material 1 [file 12889_2023_17503_MOESM1_ESM.docx]

# Online Supplement

The distribution of absences was a count variable and skewed to the right (Figure A1). So in addition to the linear models in the main document, we also fit Poisson (Table A1) and negative binomial models (Table A2) with the same fixed effects, random effects, and covariates. We fit these models using the *xtpoisson* and *xtnbreg* commands in Stata software, version 16.1

The results were very similar: a small, negative, significant main effect of small classes on absent, with a non-significant near-zero interaction between small classes and community disease prevalence. Note that Poisson and negative binomial coefficients are interpreted on a log scale, so if small classes have a coefficient of -0.06 (in the Poisson model) or -0.05 (in the negative binomial model), that means that small classes reduced absences by 5 or 6 percent.


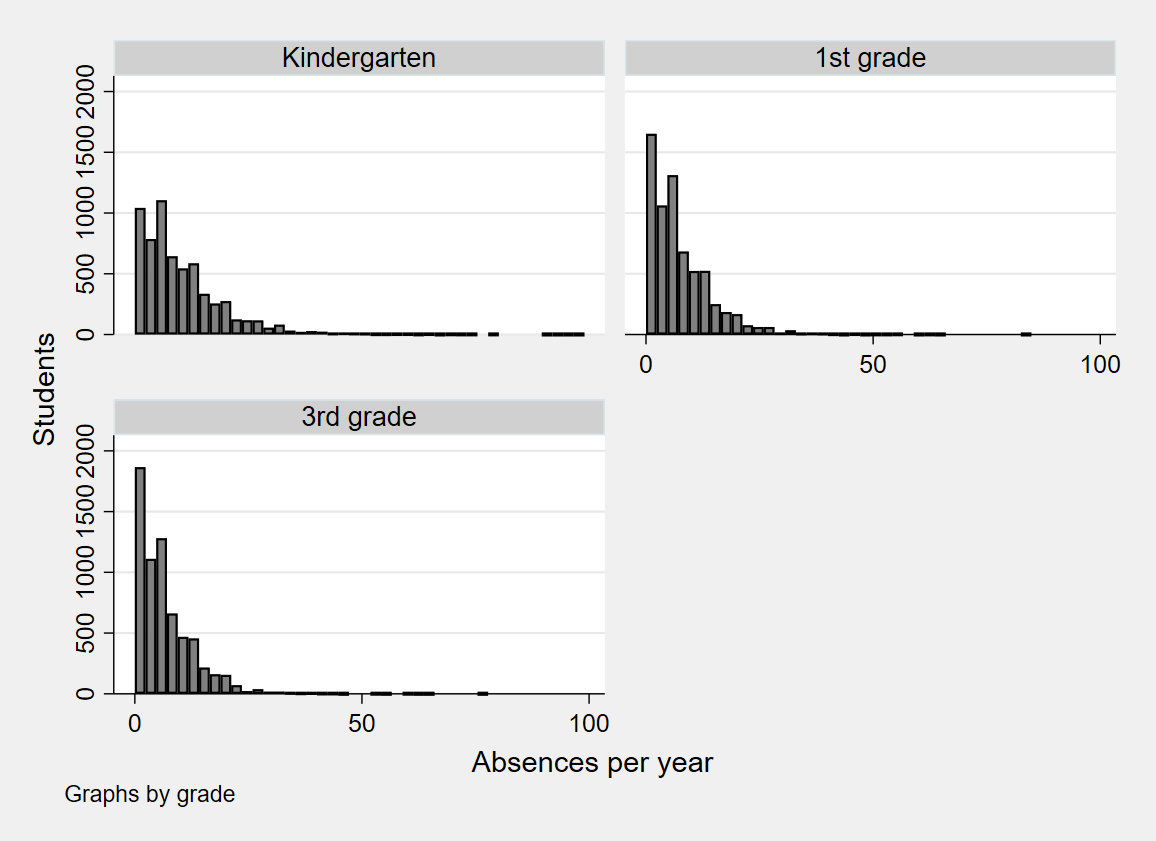


Figure A1. Distribution of student absences in kindergarten, 1^st^ grade, and 3^rd^ grade. Absences were not recorded in 2^nd^ grade.

Table A1. Poisson mixed model predicting days of absence per year, Project STAR, Tennessee, school years 1985-89.

|  | Coef.  (95% CI) | Coef.  (95% CI) | Coef.  (95% CI) | Coef.  (95% CI) |
| --- | --- | --- | --- | --- |
| Small class | -0.06* | -0.06* | -0.08+ | -0.08+ |
|  | (-0.12,-0.01) | (-0.12,-0.01) | (-0.17,0.00) | (-0.17,0.01) |
| Teacher's aide | 0.03 | 0.03 | 0.04 | 0.04 |
|  | (-0.02,0.08) | (-0.03,0.08) | (-0.04,0.12) | (-0.04,0.11) |
| First grade (ref. kindergarten) | -0.29** | -0.30** | -0.23** | -0.23** |
|  | (-0.34,-0.25) | (-0.34,-0.25) | (-0.30,-0.16) | (-0.30,-0.16) |
| Third grade (ref. kindergarten) | -0.38** | -0.38** | -0.43** | -0.44** |
|  | (-0.43,-0.33) | (-0.43,-0.34) | (-0.57,-0.29) | (-0.56,-0.31) |
| PI |  |  | 0.05+ | 0.06+ |
|  |  |  | (-0.01,0.12) | (-0.00,0.11) |
| Small class × PI |  |  | 0.00 | 0.00 |
|  |  |  | (-0.05,0.06) | (-0.05,0.05) |
| Teacher's aide × PI |  |  | 0.00 | 0.00 |
|  |  |  | (0.00,0.00) | (0.00,0.00) |
| Female (ref. male) |  | 0.03** |  | 0.02 |
|  |  | (0.01,0.05) |  | (-0.01,0.06) |
| Black (ref. white) |  | -0.14** |  | -0.11* |
|  |  | (-0.20,-0.09) |  | (-0.21,-0.00) |
| Other race/ethnicity |  | -0.20* |  | -0.31* |
|  |  | (-0.38,-0.01) |  | (-0.58,-0.04) |
| Free lunch |  | 0.12** |  | 0.16** |
|  |  | (0.08,0.16) |  | (0.08,0.24) |
| Observations | 19,499 | 19,329 | 8,073 | 8,028 |
| Distinct children | 10,816 | 10,726 | 4,966 | 4,936 |

Table A2. Negative binomial mixed model predicting days of absence per year, Project STAR, Tennessee, school years 1985-89.

|  | Coef.  (95% CI) | Coef.  (95% CI) | Coef.  (95% CI) | Coef.  (95% CI) |
| --- | --- | --- | --- | --- |
| Small class | -0.05* | -0.05+ | -0.04 | -0.03 |
|  | (-0.10,-0.00) | (-0.10,0.00) | (-0.12,0.05) | (-0.11,0.06) |
| Teacher's aide | 0.02 | 0.02 | 0.03 | 0.03 |
|  | (-0.03,0.06) | (-0.04,0.07) | (-0.05,0.11) | (-0.05,0.11) |
| First grade (ref. kindergarten) | -0.29** | -0.29** | -0.19** | -0.20** |
|  | (-0.33,-0.24) | (-0.33,-0.25) | (-0.26,-0.12) | (-0.27,-0.12) |
| Third grade (ref. kindergarten) | -0.38** | -0.38** | -0.40** | -0.41** |
|  | (-0.43,-0.33) | (-0.43,-0.34) | (-0.54,-0.26) | (-0.54,-0.29) |
| PI |  |  | 0.04 | 0.05+ |
|  |  |  | (-0.01,0.10) | (-0.01,0.10) |
| Small class × PI |  |  | -0.02 | -0.02 |
|  |  |  | (-0.07,0.03) | (-0.07,0.03) |
| Teacher's aide × PI |  |  | 0.00 | 0.00 |
|  |  |  | (0.00,0.00) | (0.00,0.00) |
| Female (ref. male) |  | 0.03* |  | 0.01 |
|  |  | (0.00,0.05) |  | (-0.03,0.04) |
| Black (ref. white) |  | -0.17** |  | -0.12* |
|  |  | (-0.22,-0.11) |  | (-0.22,-0.02) |
| Other race/ethnicity |  | -0.27** |  | -0.29* |
|  |  | (-0.43,-0.10) |  | (-0.54,-0.05) |
| Free lunch |  | 0.17** |  | 0.24** |
|  |  | (0.13,0.21) |  | (0.17,0.32) |
| Observations | 19,499 | 19,329 | 8,073 | 8,028 |
| Distinct children | 10,816 | 10,726 | 4,966 | 4,936 |
